# Is there a pilot in the brain? Contribution of the self-positioning system to spatial navigation

**DOI:** 10.3389/fnbeh.2015.00292

**Published:** 2015-10-30

**Authors:** Bruno Poucet, Franck Chaillan, Bruno Truchet, Etienne Save, Francesca Sargolini, Vincent Hok

**Affiliations:** ^1^Laboratory of Cognitive Neuroscience, CNRS and Aix-Marseille UniversityMarseille, France; ^2^Fédération 3C, CNRS and Aix-Marseille UniversityMarseille, France; ^3^Institut Universitaire de FranceParis, France

**Keywords:** landmark-based navigation, motion-based navigation, place cells, hippocampus, rat

## Abstract

Since the discovery of place cells, the hippocampus is thought to be the neural substrate of a cognitive map. The later discovery of head direction cells, grid cells and border cells, as well as of cells with more complex spatial signals, has led to the idea that there is a brain system devoted to providing the animal with the information required to achieve efficient navigation. Current questioning is focused on how these signals are integrated in the brain. In this review, we focus on the issue of how self-localization is performed in the hippocampal place cell map. To do so, we first shortly review the sensory information used by place cells and then explain how this sensory information can lead to two coding modes, respectively based on external landmarks (allothetic information) and self-motion cues (idiothetic information). We hypothesize that these two modes can be used concomitantly with the rat shifting from one mode to the other during its spatial displacements. We then speculate that sequential reactivation of place cells could participate in the resetting of self-localization under specific circumstances and in learning a new environment. Finally, we provide some predictions aimed at testing specific aspects of the proposed ideas.

## Introduction

The 2014 Nobel Prize for Physiology or Medicine was awarded to John O’Keefe, Edvard Moser and May-Britt Moser for their discovery of the “inner GPS” in the brain. Although in this formulation the analogy with human-made electronic GPS devices looks straightforward, the Nobel committee really wanted to award the discoveries of brain cells supporting higher cognitive functions including self-positioning processes. Since we all have a sense of our position in space, it seems certain that the human brain contains a neural machinery for accomplishing this function. However, the discovery of this system does not stem from research on people, but from research on animals which, over the last four decades, has revealed remarkably sophisticated coding schemes of spatial information based on the activity of specific neurons in the rat.

At the core of the system, lies the hippocampus and its place cells, which were first reported by O’Keefe and Dostrovsky (1971). Place cells are a subset of hippocampal pyramidal cells characterized by location-specific firing. As a rat moves in a familiar environment, each place cell tends to discharge only if the rat’s head is in a restricted location called the “place field”, independently of its orientation. The hippocampus contains many place cells and each place cell has a spatially distinctive place field in a given environment. Therefore, place cells are collectively signaling the current rat’s spatial location as well as the environment itself, giving rise to the possibility that they are the neural substrate of a cognitive map (O’Keefe and Nadel, 1978), that is a spatial representation of the external environment storing both spatial locations and their relationships (Tolman, 1948). This hypothesis was further strengthened in the mid-eighties by the discovery of head direction cells by Ranck (1985) (for a review, see Taube, 2007). Head direction cells fire only when the rat is heading at specific directions independently of his current location, thus providing essential and complementary information to the place cell map system. The discovery of head direction cells was also the starting point of a renewal of interest for place cells. Without this finding, place cell research could have slowly declined, making less likely the discovery of grid cells two decades later. In this sense, Jim Ranck and the Brooklyn group, who developed the appropriate tools to study head direction cell firing, should be credited for their contribution to the overall story.

The last major piece of evidence for a system dedicated to the spatial coding of the environment was provided when the existence of entorhinal grid cells was reported by the Moser’s group (Fyhn et al., 2004; Hafting et al., 2005). Contrary to place cells, grid cells have multiple place fields which show a grid-like firing pattern. They are active whenever the animal’s position coincides with any vertex of a regular grid which spans the surface of the environment. The properties of grid cells imply that they implement a directionally oriented, topographically organized neural map of the spatial environment. Furthermore, although necessarily based on self-motion cues (McNaughton et al., 2006), this map is strongly influenced by external cues and environmental geometry as well (Barry et al., 2007; Krupic et al., 2015; Stensola et al., 2015).

This very brief historical presentation illustrates how John O’Keefe’s initial observations, often judged idiosyncratic in the early years, gave rise to a vivid research field which now provides new fascinating results everyday. The current picture is a bit more complex, however, as in addition to place, grid and head direction cells described above, the computational backbone of the navigational system in the rat brain should include recently discovered border cells in the entorhinal cortex (Savelli et al., 2008; Solstad et al., 2008) and subiculum (Lever et al., 2009), place by direction cells (Cacucci et al., 2004) and grid by direction cells (Sargolini et al., 2006). Even more specific spatial signals have been found in other cell types including angular head velocity cells in the dorsal tegmental nucleus (Bassett and Taube, 2001; Sharp et al., 2001), pitch cells in the rat lateral mammillary area (Stackman and Taube, 1998) and in the presubiculum of bats (Finkelstein et al., 2015), and speed cells in several rat brain areas (medial mammillary nucleus: Sharp and Turner-Williams, 2005; interpeduncular nucleus and habenula: Sharp et al., 2006; entorhinal cortex: Kropff et al., 2015). Thus, the navigation system is widely distributed, in that its component cell types are found in several brain areas. It is thought to represent the space external to the animal in a map-like fashion, so that the rat’s position in the map is continuously updated. Ultimately, the navigation system would be used to generate optimal paths to spatial goals whatever the current position of the animal and the position of the goals within a given environment.

Although we know the main neural elements of the navigation system, we are still far from fully understanding how they work together. One central goal of ongoing research is to explain how the location, orientation and spatially periodic signals carried by each of the major cell types arise and how such signals, modified by the activity of additional cell types, permit calculations of paths through the environment. This requires establishing the links between the discharge properties of cells carrying spatial signals and the actual spatial behavior of the animal. In the present review, we focus on a single issue about hippocampal place cells. The question can be briefly summarized as follows: if place cells are a positioning system that continuously updates the rat’s position in its neural representation of the environment, how is this updating achieved during exploration and routine foraging in a familiar environment? More specifically, what is the sensory modality preferentially used by the rat to update its position? A related question is whether there is a difference in the role of place cell activity according to whether the rat simply explores the environment or navigates to specific goals. To address these issues, we first shortly review the sensory information used by place cells. We then explain how this sensory information can lead to two coding modes. We hypothesize that these two modes can be used concomitantly with the rat shifting from one mode to the other during its spatial displacements. We then speculate that sequential reactivation of place cells could participate in the resetting of self-localization under specific circumstances and in learning a new environment. Finally, we briefly conclude this review with some predictions aimed at testing specific aspects of the proposed ideas.

## Sensory Information Used by Hippocampal Place Cells

Place cells are hippocampal pyramidal cells whose firing is strongly correlated with the location of a freely moving rat in its environment. The activity of a place cell is maximal when the animal is at a particular location in the environment (the place field) and decreases as it gets further away from it. Place cells are virtually silent when the animal is outside the place field. Simultaneous recordings of large populations of place cells show that an environment is entirely mapped at a neural level and can be described as a unique spatial pattern of place fields (Wilson and McNaughton, 1993). Because there is some degree of overlapping between place fields, each location corresponds to activation of a large number of place cells. Given their strong positional firing correlates, place cells are believed to allow the animal to locate itself in its environment and to memorize different environments (O’Keefe and Nadel, 1978).

The firing of place cells is controlled by both external (allothetic) stimuli available from the environment and internal (idiothetic) information resulting from the animal’s self-motions (e.g., vestibular, proprioceptive, etc.). Virtual reality experiments in which such self-motion (vestibular) information is reduced reveals that the number of active place cells is decreased although cells still active display normal spatial firing as judged from calcium imaging (Dombeck et al., 2010) or electrophysiological unit recordings (Chen et al., 2013; Ravassard et al., 2013). Among allothetic cues, visual information seems to take precedence over other sensory modalities (for review, see Poucet et al., 2000). Thus, rotation of remote visual cues in a given environment induces equivalent rotation of place fields (O’Keefe and Conway, 1978; Muller and Kubie, 1987). Furthermore, when salient visual landmarks are removed or when the lights of the recording room are switched off while the animal is running in a familiar environment, place cells continue to fire in the original location (Muller and Kubie, 1987). This indicates that, to some extent, self-motion information is sufficient to maintain field activity in the absence of allothetic cues (Save et al., 1998; Zhang et al., 2014).

To use self-motion information, the rat would update its position by tracking changes in position using signals derived from a variety of sources including vestibular cues (Wiener et al., 1995), visual motion cues such as optic flow (Sharp et al., 1995) and proprioceptive cues (McNaughton et al., 1996). In theory, any combination of self-motion information would allow the rat to update its position as it moves in space. In practice, the iterative nature of the path integration process leads to errors which accumulate with increasing distance traveled and increasing time (Etienne and Jeffery, 2004). Difficulties with pure self-motion based positioning are confirmed by the finding that place cell discharge becomes spatially unreliable (i.e., drifts or becomes silent) when external cues are dramatically restricted (Save et al., 2000), which suggests that the total error is so large that self-localization becomes impossible.

Since self-motion information allows only limited navigation performance due to error accumulation, there must be a recalibration process that corrects errors in the calculated rat’s position. Presumably such recalibration involves the gathering of information from many different sensory systems but visual information, when available, plays a key role in this process (e.g., McNaughton et al., 1991). This is because vision allows for collecting, at a distance, a large amount of spatial information that is both precise and holistic (i.e., cue configurations rather than discrete local cues are encoded), thus enabling organisms to cope rapidly with the major features of their environment. Recent evidence however indicates that place field stability in darkness may also be sustained by border information, i.e., when the animal encounters the environmental boundaries of explored space (Zhang et al., 2014). A recent developmental study by Muessig et al. (2015) also suggests that place cell representations are more stable and more accurate close to environmental boundaries in pre-weanling rats, i.e., when distal landmarks are not accessible due to the immaturity of the visual system in rat pups. Because at this stage stable border cells are available, this finding suggests that environmental boundaries may be used to calibrate the place cell via boundary-responsive neurons such as border cells. It is only later, after weaning, that place cells become equally stable and accurate throughout the environment. This developmental switch in place cell accuracy coincides with the maturation of the entorhinal grid cell network, raising the possibility that grid cells contribute to stable place fields when an organism is far from environmental boundaries. Nevertheless, it is interesting to observe that grid cell firing fields may themselves drift, for example in mice exploring large arenas in the light (Hardcastle et al., 2015). That these drifts appear to be corrected by encounters with environmental boundaries (Hardcastle et al., 2015; see Cheung et al., 2012 for theoretical elaboration of this process) points out the need of a recalibration process for space coding systems (even in the light) and the importance of salient landmarks (here the boundaries) in this process.

There are two ways in which the recalibration process may operate. First, it may solve major and sudden discrepancies in the hippocampal map orientation. A good example of this process is provided by the study of Rotenberg and Muller (1997). In this work, hippocampal place cells were recorded as a salient visual cue in the recording arena was rotated while the rat was *in the apparatus*. This manipulation puts into conflict visual stimuli (which indicate that the surroundings have moved) with self-motion stimuli (which indicate the surroundings are stable). Rotenberg and Muller (1997) found that if the card was rotated by a small angle (45°), fields almost always rotated equally whereas if the card was rotated by 180° the fields almost always remained in their previous position. This effect is due to the conflict between external and self-motion cues. If the card rotation is not too great, the magnitude of the discrepancy is consistent with a small error in self-motion calculations. Therefore, the system relies preferentially on the external environment and updates the angular reference frame for place cells. If the card rotation is large, the discrepancy is too large to be consistent with a self-motion error because such errors are the result of a progressive build-up with movements and are unlikely to occur so abruptly. As a consequence, the angular reference frame for place cells needs not be changed.

The recalibration may also operate more continuously as the rat moves in the recording environment. An illustration of this process is a study in which place cells were recorded from blind animals. In these rats, place fields were perfectly stable relative to a set of objects in the recording arena, even when they were located away from the objects (Save et al., 1998). That the system was able to compute a position everywhere in the environment, and not just at object locations, implies that it relied on the dynamic “online” use of self-motion cues to update the rat’s position throughout the environment, in particular at locations away from the objects. In-depth behavioral analysis revealed how the coherence of the map was maintained in spite of the rat mainly relying on self-motions. Indeed, the behavior of blind rats revealed that they used the fixed object locations for recalibrating calculated positions by making more frequent exploratory contacts with the objects than sighted rats. This “compensatory strategy” provides blind rats with the information required to recalibrate their position in the arena. It is likely that a similar process occurs in sighted rats although probably visually based.

To sum up, experimental evidence shows that the hippocampal map relies on both external/allothetic or internal/idiothetic cues. The use of either cue type depends on its availability and reliability. Furthermore, idiothetic (self-motion) cue processing requires a recalibration process to correct for its inherent errors.

## Two Self-Localization Modes Possibly Implemented by Neural Activity Switches

Although, as shown above, it is experimentally possible to require the rat to use allothetic or idiothetic cues, the two types of cues are simultaneously available in most situations and offer complementary information. External landmarks allow precise localization of the rat’s current position and of distal locations. In contrast, self-motion cues allow ongoing calculation of the rat’s current position in a short range. The main asset of relying on self-motion cues is that it reduces the need to pay attention to external cues. Therefore, self-motion information presumably reduces the cognitive burden on the navigational system imposed by dealing with external spatial cues, even though the rat must occasionally recalibrate its position to correct for errors on the basis of allothetic cues. Arguably, this could leave more resources for processing other information (Poucet et al., 2000).

It is therefore reasonable to assume that, even when it is not absolutely required to use self-motion cues, such information will actually be automatically integrated to allow the rat to perform ongoing calculations of its position. In this view, animals track their position in a framework provided by landmark stimuli or by using self-motion information. Pure self-motion navigation cannot remain accurate over indefinite distances or times; discrepancies between the computed and actual positions will accumulate unless a landmark-based resetting mechanism can put computed position back into register with the true position. Thus, self-motion navigation ultimately requires landmark references. Processing of stable external cues would occur during specific episodes in the course of spatial exploration (i.e., for positional resetting) or during navigation for computing goal-directed paths. We believe that in a familiar environment the rat’s spatial navigation system constantly switches between the two navigational strategies.

Neural evidence for such a switching process is provided by the recent demonstration that variability in place cell firing is strongly modulated by the attentional requirements of the task (Fenton et al., 2010). Fenton and Muller (1998) were the first to demonstrate that place cell firing during individual passes through the place field is less reliable than predicted by the overall averaged activity, a phenomenon called “overdispersion”. Subsequent observations by Olypher et al. (2002) and Jackson and Redish (2007) suggested that overdispersion reflects the switch between different states possibly corresponding to different cognitive representations of the environment.

In a more recent analysis, overdispersion was measured in several conditions differing by the task requirements (Fenton et al., 2010). The general hypothesis was that overdispersion reflects discharge fluctuations that result from attentional switches between distal cues and local/self-motion cues. This analysis revealed that overdispersion was reduced during goal-directed navigation relative to random foraging and that the use of one set of cues (i.e., distal vs. local/self-motion cues) during navigation further decreased it. Furthermore ensemble discharge could be separated into two time-dependent attentional states during which firing of individual cells was substantially different. The authors proposed that place cell activity is modulated by a behaviorally silent attention-like process, likely under the influence of the prefrontal cortex (Hok et al., 2013), which spontaneously switches between distinct ensemble place codes. This dynamic process would modulate cell discharge on a timescale of approximately 1 s, with each place cell code representing currently attended spatial information. The advantage of such dynamical coding is that it guarantees accurate self-localization while maintaining available attentional resources toward potentially important information.

## Possible Basis for the Two Self-Localization Modes

Because our proposal entails that both external and self-motion cues are used during self-localization, it is expected that damaging brain areas in charge of their processing will result in alterations of place cell firing. Subtle alterations are indeed found following a wide variety of brain lesions (for a review, see Poucet et al., 2003). Unfortunately it is difficult to appreciate the impact of lesions on the use of the two classes of cues as they are selectively manipulated only in a few studies^1^. There are however a few noticeable exceptions.

First, both the parietal and retrosplenial cortices appear to contribute to the integration of visuo-spatial and motion-related cues for navigation (Mizumori et al., 2000; Save and Poucet, 2000a). Their lesion induces deficits in spatial tasks and alter hippocampal place cell activity in specific ways. For example, inactivation of the retrosplenial cortex impaired initial learning of the radial arm maze task, and impaired retention of learning only when the animals were tested in darkness. These deficits were associated with a loss of place field stability (i.e., the location of place fields shifted to unpredictable locations of the maze), thus suggesting the involvement of the retrosplenial cortex in the integration of allothetic and idiothetic information (Cooper and Mizumori, 2001). Contrary to retrosplenial inactivations, parietal cortex damage did not impair place field stability in standard conditions nor did it alter control by the objects landmarks present in the recording arena (Save et al., 2005). The most dramatic effect of parietal lesions was observed when the object landmarks were removed in presence of the animal, as a majority of fields shifted back to the initial pre-rotation location, suggesting that they were under control of background cues. This raises the possibility that the parietal cortex is involved in the processing of proximal object landmarks (see also Save and Poucet, 2000b), and may contribute to provide a stable local reference frame to place cells by using the objects as an anchor system to reset self-localization based on motion cues. In this interpretation, parietal-damaged rats would be unable to use self-motion cues to maintain place field stability in absence of objects and would rather rely on uncontrolled background information.

Entorhinal damage yields a slightly different outcome following landmark removal (Van Cauter et al., 2008). Although, as expected, place fields in rats with entorhinal damage are unstable compared to controls when landmarks are removed, there is no trend for them to align with static uncontrolled background cues. In other words, their new angular position is randomly anchored in space as if, in addition to a difficulty in using self-motion cues, there was an additional loss of directional information provided by the distal environment.

Even though specific effects of cortical damage are observed on how landmark information gets integrated with motion cues, there is no strong evidence for a major contribution of any single cortical area so far studied, with the exception of the retrosplenial cortex which may play a more important role. It is however possible that such integration is under joint control of several pathways. In that case, there could be a brain target whose role is to put together information carried by these pathways. One candidate structure for this function is the postsubiculum, a part of the head direction system which projects directly to the entorhinal cortex. In a comprehensive review of the visual stream pathways that may carry information about landmarks, Yoder et al. (2011) conclude that the postsubiculum is in a key anatomical position for integrating landmark and motion-related information. Visual information reaches the postsubiculum through several pathways, including a dorsal visual stream (via parietal and retrosplenial cortex), a ventral visual stream (via postrhinal/perirhinal and retrosplenial cortex), but also a more direct stream bypassing the above mentioned structures. On another hand, motion-related information reaches the postsubiculum via several nuclei containing head direction cells, in particular the dorsal tegmental nucleus and lateral mammillary nuclei which receive vestibular, proprioceptive and motor information (Yoder et al., 2011). Interestingly, lesions to the postsubiculum have detrimental effects on landmark control in both hippocampal place cells (Calton et al., 2003) and anterodorsal thalamic head direction cells (Goodridge and Taube, 1997). Because postsubicular head direction signals are sent to the entorhinal cortex, they may be essential to the emergence of grid cells where landmark and motion-related information also appears to be integrated.

Based on the above evidence and the properties of grid cells, we recently proposed the following scenario to account for the interplay between allothetic and idiothetic cues in self-localization (Poucet et al., 2014). Briefly, we assume that self-localization is accomplished by hippocampal place cells, whether it is based on landmark or motion-related information. Both streams allow to move the rat’s calculated position in the hippocampal map. However, entorhinal grid cells have a crucial navigational role only in the dark when their activity is dictated by self-motion cues and controls place cell activity. Their role is to provide a means by which place cells can track the animal’s location using only self-motion information, when absolute sensory information is unavailable, degraded or ignored. When possible, however, grid cell activity is updated by external sensory information to reduce the accumulating odometric error. In the light, properly scaled spatial computations can be carried out, in principle, by the hippocampus without assistance from entorhinal grid cells. Furthermore grid cell activity is mainly shaped by visual stimuli in the light even though self-motion cues are still integrated. Although our model made other assumptions about the functional connectivity between the entorhinal cortex and the hippocampus, the very brief scenario outlined above is sufficient to grasp the core notions of our proposal.

## Self-Localization and the Cognitive Map

### Place Cells and Exploration

Self-localization is only one of the functions of the spatial map implemented by hippocampal place cells. If the ultimate function of the map is to support spatial navigation, this function relies first on appropriate coding of the current environment. If such coding is not accomplished, the animal must explore the environment so that its map matches real space as closely as possible (Poucet, 1993). The neural consequence of encoding new information about the environment is reflected in the phenomenon of remapping, in which the activity of place cells is changed. Remapping can be global or local. Global remapping is observed when the whole subset of cells active in one environment is changed when the rat is introduced in another environment. It therefore reflects a pattern separation process in which two environments are coded by two orthogonal representations.

In contrast, local remapping is observed when the activity of only a fraction of the active place cells is modified (for a review, see Muller et al., 1999). This happens for example when objects are repositioned within an otherwise familiar arena (Lenck-Santini et al., 2005) or when subtle changes in the environment alter its topological structure (Alvernhe et al., 2008, 2011). In general local remapping is specific to the locus of the change. Arguably, therefore, local remapping would reflect updating of the topological representation of the environment (Alvernhe et al., 2011; Dabaghian et al., 2014). This suggests that place cells code more than just the animal’s spatial location, but provide also information about the relationships between locations, and thus possible paths. Furthermore, because any topological change results in both visual and motoric effects, it is likely that local remapping relies on both landmark and self-motion information. Although there is only scarce evidence of how new information is detected and integrated by place cells, two recent studies provide some hints as to possible mechanisms. The first study found that increased neural activity during exploratory head-scanning behaviors predicted the formation and potentiation of place fields on the next pass through that location (Monaco et al., 2014). This rapid potentiation of place fields coordinated by scanning behavior was interpreted as reflecting the incorporation of information about attended external stimuli onto the hippocampal spatial framework. In the second study, detection of both spatial and non-spatial novelty was found to be associated with robust increases in firing rate in CA1, but not in CA3 (Larkin et al., 2014). These firing rate increases persisted during sharp wave ripples, when place cell representations of previous experiences were replayed, suggesting that detected novelty was integrated and stored in the hippocampal representation.

### Place Cells and Navigation

As mentioned above, the ultimate function of the spatial map is to permit efficient navigation to potential goals. The involvement of place cells in path planning is supported by recent findings showing that they may encode paths forward of the animal through a mechanism of sequential reactivations (Johnson and Redish, 2007; Pfeiffer and Foster, 2013). Although sequential reactivation were initially reported to occur during sleep, it was also observed to occur in the awake state during periods of relative immobility (Skaggs and McNaughton, 1996; Foster and Wilson, 2006; review in Buhry et al., 2011; Carr et al., 2011). During “replay”, hippocampal place cells that fired during exposure to an environment are orderly reactivated at a subsequent time so that the initial experience is recapitulated over a very brief period of about 50–100 ms, a phenomenon taken to reflect the operation of an offline consolidation mechanism (e.g., Girardeau et al., 2009; but see Gupta et al., 2010 for counter-evidence). However, place cells were more recently reported to sequentially activate for locations not yet experienced in wakefulness (i.e., “preplay”, Dragoi and Tonegawa, 2011). These activations were seen during an EEG state characterized by high-frequency oscillations, i.e., sharp waves ripples (SWRs). With regard to the relationship between navigation and place cell reactivations, Johnson and Redish (2007) found place cells to transiently reactivate at choice-point where the rat had to make a decision in such a way that the location reconstructed from the neural ensemble swept forward, first down one path and then the other. Such transient activations are a form of preplay in that coherent representations were preferentially swept ahead of the animal rather than behind the animal. Presumably, they represent future possibilities, as if the rat was assessing the two alternatives choices. Even more convincing evidence for the involvement of place cells in navigation is the recent finding of a flexible, goal-directed mechanism, in which behavioral trajectories to a remembered goal are depicted in the brain immediately before movement (Pfeiffer and Foster, 2013; see also Singer et al., 2013). In an open arena, the rat hippocampus was shown to generate brief sequential activations of place cells that predicted the immediate future path taken by the rat to its spatial goal.

### A Possible Role for High-Frequency Oscillations in Self-Localization

Sequential reactivations such as replay and preplay occur during high-frequency oscillations (Buzsáki, 1989). Although evidence suggests that sequential reactivations during sleep play a functional role in consolidation (Carr et al., 2011), they may reflect more than just the fixation of recent experience in memory (Gupta et al., 2010). Reactivations in the awake state are even more difficult to interpret along this line as they differ in several ways from sleep reactivations (Buhry et al., 2011). Furthermore, the mere existence of preplay is difficult to reconcile with the notion that sequential reactivations correspond strictly to a consolidation process. Thus the intriguing possibility exists that high-frequency oscillations and replay reflect more than just consolidation of immediate past events. Other hypotheses about replay include detection of novelty, modifying neural representations, attention, planning, motion, and memory retrieval (Buhry et al., 2011).

Returning to self-localization, we would like to propose the following speculative account of how high-frequency oscillations and the associated phenomena of replay and preplay might have a specific role. First, as explained in section “Two self-localization modes possibly implemented by neural activity switches”, we imagine that in the light the rat tracks its position by switching between using landmark information and self-motion information. Overdispersion would reflect these two coding modes. As landmark information is constantly available, the resetting process which allows correction of self-motion based navigation by recalibration based on allothetic cues would operate continuously and at a fast rate, i.e., every one second (Fenton et al., 2010), thus preventing any marked drift of self-localization.

**Figure 1:**
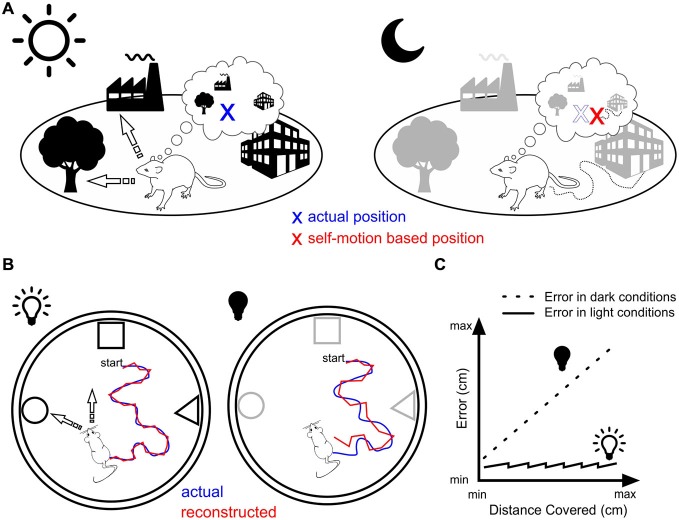
**Landmark vs. motion-based navigation. (A)** Rat navigating in daylight (left) can orient itself by triangulating its location (blue cross) based on bearings to two or more known landmarks (arrows). The same rat in darkness (right) that would rely solely on self-motion navigation would miscalculate its position (red cross) due to the accumulation of error. **(B)** Experimental assessment of the position error generated by self-motion navigation. In light conditions (left), the animal is thought to use different strategies to orient itself (e.g., triangulation and path integration) leading to minor errors between the actual position (blue line) and the reconstructed spatial trajectory of the animal as estimated from the place cells’ population firing rate vector (red line, e.g., Wilson and McNaughton, 1993). In dark conditions (right), no visual recalibration can be operated leading therefore to an increase in the error between actual and reconstructed trajectories. **(C)** In the absence of physical contact with the objects or the borders of the arena, this error might increase linearly as the distance travelled increases (dotted line), while it is reset periodically in light conditions (plain line) through attentional shifts (overdispersion) or ripples activity.

In contrast, resetting in the dark is possible only if the rat physically encounters a familiar landmark cue. The frequency at which this happens depends on a variety of factors including the animal’s speed, the size of the environment, the number and distance between landmark cues. Under these circumstances, the resetting process must operate at a rate that is a trade-off between two opposite requirements, namely keeping self-localization as accurate as possible on the basis of self-motion cues and on the other hand, maintaining a certain level of locomotion activity. In other words, resetting should occur for a travelled distance that is below the point at which self-localization would be at odds with the rat’s current location (Figure 1).

In experimental setups, one convenient way of controlling access to allothetic cues is to turn off the lights, while leaving one or more salient objects at well-defined positions in the arena so that they can serve as resetting landmarks. In this way, it becomes possible to measure the changes in behavior and place cell firing and see how the rat recalibrates its position at the object locations. Amazingly, however, this experiment has not been done so far. Another way of controlling access to allothetic cues is to deprive the rat from visual information. The study by Save et al. (1998) followed this idea by recording place cells from blind rats. It revealed that these rats contacted the objects more frequently than sighted rats, which suggests that they used the fixed object locations for resetting motion-based calculated position. There is also evidence that such resetting occurs for head direction cells as well (Valerio and Taube, 2012). In this study, the authors recorded anterodorsal thalamic head direction cells as blindfolded rats had to perform a food-carrying path integration task (Whishaw and Tomie, 1997). They found a robust correlation between head direction cell activity and rat’s heading errors during path integration-based homing behavior. Furthermore, after small amplitude angular shifts (the most frequent case), head direction cell’s preferred firing directions were reset to stable values anchored to the homing base whereas after large amplitude angular shifts (much less frequent), the head direction system was observed to take a new bearing which was usually stable for subsequent trials (Valerio and Taube, 2012). It is difficult to know what place cells would do in the later circumstances, but one likely possibility is that they would collectively rotate their place fields to stay in register with the head direction compass.

Because such resetting requires to put in register past information with current information, our speculation is that it is associated with the replay of the path that led the rat to the salient landmark object used to anchor the place representation. In this view, the sequential reactivation of place cells, which represent previous trajectories on a compressed time scale, provides the putative mechanism through which the calculated position can be compared with the actual outcome of the rat’s path so that it can be corrected. Interestingly, the same mechanism could be operating during initial learning of a new environment so that self-motion information becomes “linked” to landmark information in the hippocampal map (McNaughton et al., 2006).

In line with this last idea, the creation of such “compound firing fields” (*i.e*., firing fields depending on both self-motion and landmark information) could rely on theta sequences supported by the medial septum activation. Indeed, a recent study by Wang et al. (2015) showed that septal inputs were required to initiate and maintain hippocampal place fields generated while the animal was running in a wheel. These so-called “internally generated firing fields” were abolished by septal inactivation even in highly familiar situations while the firing fields present in the maze arms remained unaffected. Additionally, this study showed that some firing fields did not form in a novel environment in the absence of septal inputs but were maintained under septal inactivation once they were well established. Taken together, the authors interpret their results as evidence that theta sequences are instrumental in shaping hippocampal place field activity while the rat is engaged in an episodic-like memory task. However, one cannot rule out the possibility that these internally generated place fields reflect a more simple integration of self-motion generated cues by the hippocampus. This hypothesis is further reinforced by the fact that grid cell activity, which has been suggested to support path integration, is disrupted by septal inactivation (Brandon et al., 2011; Koenig et al., 2011) and during passive transport (Winter et al., 2015). Therefore, the role of theta sequences, through septal activation, could be interpreted as a mechanism to organize and implement self-motion cues into the hippocampal representation.

## Conclusion

Among the issues that, in our opinion, are important to be addressed to explain how spatial navigation can be shaped by hippocampal activity, how the animal keeps track of its position in the current environment is a central question. Here, we assume that the rat’s position in space is coded in the activity pattern of hippocampal place cells and we hypothesize that the dynamics of such coding is continuously maintained by two complementary sources of information, namely landmark cues and self-motion cues. Because both types of cues are available in most circumstances and have complementary assets, we believe that they are used concurrently. Thus, in the course of a journey self-motion cues are automatically integrated so as to track the animal’s position in the hippocampal map. As pure self-motion navigation is not accurate, a landmark-based mechanism resets animal’s position in the place cell representation. The switch between the two strategies would manifest itself through the phenomenon of overdispersion, in which the existence of two slightly offset hippocampal maps, each under the influence of a specific class of cues, would appear as two distinct hippocampal states (Jackson and Redish, 2007; Fenton et al., 2010). So far, no clear overt behavior has been shown to be associated with such switches. Similarly the neural mechanisms are still unknown. However, we speculate that one possible candidate could be the replay phenomenon, which would allow comparing the rat’s position calculated on the basis of self-motion cues with its actual position based on landmark information.

One way to test these ideas is to record place cell activity as the rat explores an arena providing discrete object landmarks in the dark. Because the spatial information provided by such objects is local, only direct physical contacts with them would allow the rat to use them to reset its positioning system. Thus, if the above scenario is true, we predict the following outcomes. First, the number of contacts with the objects and/or with the borders of the arena should increase to help resetting self-localization in the dark. Since the path integrator based on self-motion cues is challenged to a greater extent in the dark compared to the light, the only way for the rat to recalibrate its position is to collect information from the object landmarks and environmental boundaries, which should lead to increased frequency of contacts. Second, because self-motion based navigation is thought to depend on grid cell input to the hippocampus (Poucet et al., 2014), damage to the medial entorhinal cortex should impair the processing of self-motion information, and therefore should result in a further increase of the number of contacts with the object landmarks so as to recalibrate self-localization even more frequently. Third, the number of replay episodes and SWRs associated with exploratory contacts with the objects should be greater in the dark than in the light. Again this would occur because resetting can be done everywhere when based on visual landmarks, while it can occur only at object locations in the dark. If replay is the hallmark of the resetting process, then it should increase at object locations when the rat is tested in the dark. Fourth, if theta sequences are of particular importance in shaping place field activity based on self-motion cues, inactivation of the medial septum, which disrupt theta generation, should result in profound disruption of hippocampal place cell activity in darkness but not in the light (Wang et al., 2015). Lastly, animals with damaged medial entorhinal cortex should express less reliable replay as a consequence of impaired self-motion processing, particularly in the dark.

Recent progress in understanding the physiological and mechanistic aspects of the navigational system in the brain is tremendous. However, it is our contention that the detailed understanding of the behavioral mechanisms underpinned by these brain processes has not been the subject of so much attention. Overall, we think that there is a deep need for re-assessing the fine structure of the spatial behavior of the animal in register with the discharge properties of cells carrying spatial signals. A good illustration of the type of analysis that would be useful is provided by studies in which neuronal activity is analyzed in relation to specific behaviors at specific places, such as decision points (e.g., Johnson and Redish, 2007; Catanese et al., 2012), specific landmark objects (e.g., Save et al., 1998), goal locations (e.g., Hok et al., 2007), as well as during performance of well-designed spatial navigation tasks (e.g., Pfeiffer and Foster, 2013). In such a way, it becomes possible to draw functional relationships between cell firing and cognitive processes hypothesized to support self-localization and navigation. Although such correlational approach needs to be complemented by analyses of causal relationships, we believe that it is only through a detailed description of actual spatial behavior that we can understand how spatial navigation, which looks so simple at first sight but is so complex in reality, can be implemented by the coordinated activity of a widespread brain system.

## Author Contributions

All authors discussed the ideas expressed in this review. BP and VH wrote the article and all authors participated in editing and improving its different versions.

## Conflict of Interest Statement

The authors declare that the research was conducted in the absence of any commercial or financial relationships that could be construed as a potential conflict of interest.

## Footnotes

^1^In addition, while it is relatively easy to manipulate external cues (e.g., by recording place cell activity after selective cue removal, or in total darkness, etc.), it is much more challenging to neutralize the use of idiothetic information without jeopardizing the mere existence of place cells (virtual reality systems are helpful nonetheless in helping to solve this specific issue).
